# Control of anisotropic conduction of carbon nanotube sheets and their use as planar-type thermoelectric conversion materials

**DOI:** 10.1080/14686996.2021.1902243

**Published:** 2021-04-13

**Authors:** Masamichi Matsumoto, Ryohei Yamaguchi, Keisuke Shima, Masakazu Mukaida, Motohiro Tomita, Takanobu Watanabe, Takao Ishida, Tsuyohiko Fujigaya

**Affiliations:** a Department of Applied Chemistry, Graduate School of Engineering, Kyushu University, Nishi-ku, Japan; b Faculty of Science and Engineering, Waseda University, Shinjuku-ku, Japan; c Nanomaterials Research Institute, Department of Materials and Chemistry, National Institute of Advanced Industrial Science and Technology, Tsukuba, Japan; d Research Institute for Ambientornics, Waseda University, Shinjuku-ku, Japan; e Global Zero Emission Research Center, National Institute of Advanced Industrial Science and Technology, Tsukuba, Japan; f International Institute for Carbon Neutral Energy Research, Kyushu University, Nishi-ku, Japan; g Center for Molecular Systems, Kyushu University, Nishi-ku, Japan

**Keywords:** Carbon nanotubes, thermoelectric conversion, anisotropy, thermal conductivity, electrical conductivity, polymer particle, sacrificial template, wearable battery, 50 Energy Materials, 102 Porous / Nanoporous / Nanostructured materials, 104 Carbon and related materials, 210 Thermoelectronics / Thermal transport / insulators

## Abstract

The large anisotropic thermal conduction of a carbon nanotube (CNT) sheet that originates from the in-plane orientation of one-dimensional CNTs is disadvantageous for thermoelectric conversion using the Seebeck effect since the temperature gradient is difficult to maintain in the current flow direction. To control the orientation of the CNTs, polymer particles are introduced as orientation aligners upon sheet formation by vacuum filtration. The thermal conductivities in the in-plane direction decrease as the number of polymer particles in the sheet increases, while that in the through-plane direction increases. Consequently, a greater temperature gradient is observed for the anisotropy-controlled CNT sheet as compared to that detected for the CNT sheet without anisotropy control when a part of the sheet is heated, which results in a higher power density for the planar-type thermoelectric device. These findings are quite useful for the development of flexible and wearable thermoelectric batteries using CNT sheets.

## Graphical abstract

**Figure:**
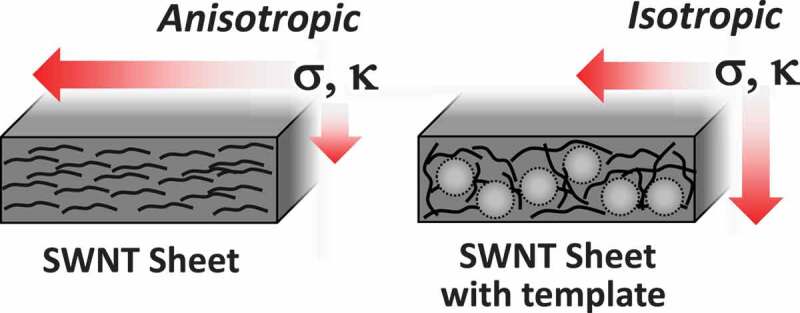

## 1. Introduction

A carbon nanotube (CNT) sheet is a promising material for various applications, such as energy storage and fabrication of electrodes and actuators, owing to its excellent electrical conductivity, flexibility, and mechanical toughness [1]. Thermoelectric (TE) conversion is one of the key applications for a CNT sheet, in which an electrical current is generated based on the temperature gradient (the so-called Seebeck effect); this is particularly due to its remarkable electrical conductivity [2,3]. It has been demonstrated that a CNT sheet possesses large conductivity anisotropy, and its electrical conductivity (σ) in the in-plane direction (1.51 × 10^3^ S cm^−1^) is 20 times more than that in the through-plane direction (65 S cm^−1^) [4]. Therefore, for TE conversion, the in-plane direction has been used for the current generation [3,5]. Similarly, high anisotropic thermal conductivity (*κ*) is exhibited, and the *κ* in the in-plane direction (9.8 ± 3.3 Wm K^−1^) is 70 times more than that in the through-plane direction (0.13 ± 0.001 Wm K^−1^) [4]. Such large anisotropic conductivities of CNT sheets are unfavorable for maintaining a temperature gradient in the current flow direction. Meanwhile, isotropic CNT sheets, which are fabricated by the chemical vapor deposition method known as ‘CNT 3D sponges,’ [6] have been reported to exhibit isotropic *κ* values as low as 0.035 W mK^−1^. Since this approach led low σ value (120 S cm^−1^), control of the in-plane thermal conductivity with a minimal decrease in electrical conductivity has been required.

Here, we demonstrate the control of anisotropy using spherical polymer particles as orientation aligners of CNT. When polymer particles are incorporated into a CNT network, the CNTs surround the particle surfaces and their orientation is altered. The degree of change in the orientation can be controlled by varying the amount of polymer added to the CNT sheet. The in-plane and through-plane thermal and electrical conductivities and Seebeck coefficients of the CNT sheets varied according to the amount of spherical polymer particles added. Heat diffusion of the CNT sheets with and without anisotropy control was monitored and the results show that the temperature gradient becomes larger when the orientation of the CNT is controlled.

## 2. Experimental

### 2.1. Materials

Single-walled carbon nanotubes (SWCNTs; eDIPS EC1.5) with a diameter of 1.5 ± 0.5 nm were purchased from Meijo Nano Carbon. Methanol was purchased from Kanto Chemical. Sodium dodecylbenzenesulfonate (SDBS) was purchased from Tokyo Chemical Industry and used as received.

### 2.2. Measurements

X-ray photoelectron spectroscopy (XPS) was conducted using an AXIS-ULTRA system (Shimadzu, Kyoto Japan), in which indium was used as the substrate. Thermogravimetric analysis (TGA) was carried out using a TG/DTA 6300 instrument (Hitachi High-Tech, Tokyo Japan) at a heating rate of 10 K min^−1^ under flowing air (150 mL min^−1^). Scanning electron microscopy (SEM) was carried out using a SU-9000 microscope (Hitachi High-Tech, Tokyo, Japan) with an acceleration voltage of 10 kV. The in-plane electrical conductivity and Seebeck coefficient were determined using a ZEM-3M measurement device (ADVANCE RIKO, Yokohama Japan) under helium at a reduced pressure (0.01 MPa). The through-plane electrical conductivity and Seebeck coefficient were measured using a homemade system, where a programmed temperature gradient was applied between the top and bottom of the sheets. Differential scanning calorimetry (DSC) was carried out using an EXSTAR DSC 6220 instrument (Hitachi High-Tech, Tokyo Japan) at a heating rate of 10 K min^−1^ under flowing nitrogen (100 mL min^−1^). The specific heat capacity (C_p_) was calibrated using a sapphire crystal (Al_2_O_3_). The in-plane and through-plane thermal diffusivities were measured using a Thermowave Analyzer TA (Bethel, Ishioka Japan). The density was determined from the weight and volume of the sheets. Temperature mapping of the sheet surface was recorded using TVS-500EX equipment (Nippon Avionics, Yokohama Japan). The mechanical properties were measured using a micro-compression tester MCT-510 (Shimadzu, Kyoto Japan).

### 2.3. Fabrication of SWCNT sheets

Five hundred milligrams of SDBS in deionized water (100 mL) was added to SWCNTs (10 mg) and then stirred for 15 min, followed by bath-type sonication for 60 min. After sonication, PS particles (Polysciences, Inc., diameter: 3.0 µm) were added through dispersion (1, 10, 50, 100, and 150 mg) and the mixture was filtered to create the SWCNT sheet containing PS. After drying at 80°C for 8 h, the SWCNT sheet was dipped in ethanol for 24 h to remove the SDBS and then heated at 450°C for 1 h to remove the PS particles.

**Scheme 1.**
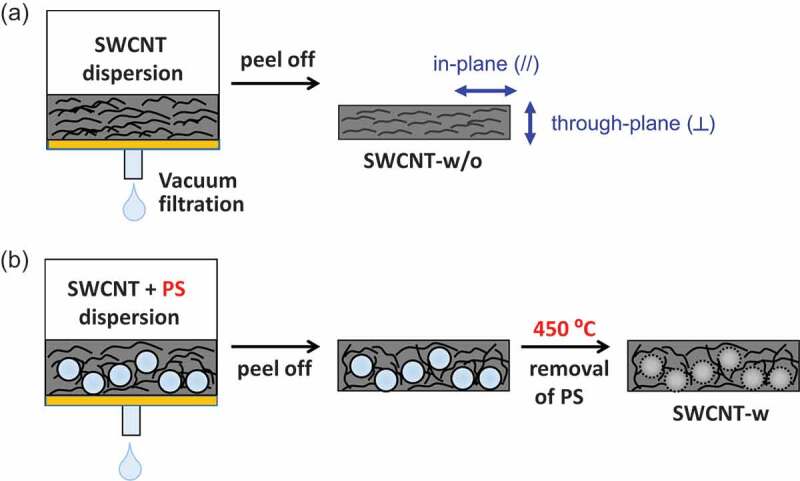
Preparation of the SWCNT sheet (a) without and (b) with anisotropy control

### 2.4. Monitoring of thermal diffusion

SWCNT sheets with thermal insulating layer (TIL) and copper wire were placed on a temperature-controlled hot plate heated at 40°C. Changes in temperature were monitored by an IR camera, and the temperature distribution was recorded after 2 min because no prominent change in temperature distribution was observed. The TIL was prepared by adding 15 mL of silica aerogel (ENOVA, IC3110) to a 3 wt% poly(vinyl alcohol) (PVA) aqueous solution (15 mL) and stirring for 1 h [7].

### 2.5. Thermal diffusion simulation

The temperature distribution of the SWCNT sheet was simulated using infinite element simulation using COMSOL Multiphysics software (version 5.1). Thermal conductivities of 400 and 0.003 W m^−1^ K^−1^ were used for the Cu and silica aerogel/PVA composite, respectively, for the simulation.

## 3. Results and discussion

### 3.1. Control of single-walled carbon nanotube anisotropy

Thermal conductivity (*κ*), electrical conductivity (σ), and Seebeck coefficient (S) of the single-walled carbon nanotube (SWCNT) sheet in the in-plane (//) and through-plane (⊥) directions prepared by the filtration method (Scheme 1(a)) were measured. It was found that *κ*_//_and *κ*_⊥_ were 25.9 and 0.0874 Wm K^−1^, and σ_//_and σ_⊥_ were 3.96 × 10^4^ and 1.56 S cm^−1^, respectively (Table S1). Large conductivity anisotropies that are over 300 and 2.5 × 10^4^ times for the thermal (*κ*_//_/*κ*_⊥_) and electrical (σ_//_/σ_⊥_) conductivities, respectively, have been observed. Since SWCNTs possess a one-dimensional structure and tend to lie in the in-plane direction upon filtration, conduction is more favorable in the in-plane direction [4]. Similar conductivity anisotropy has also been reported for semiconducting polymer films [8].

**Figure 1.**
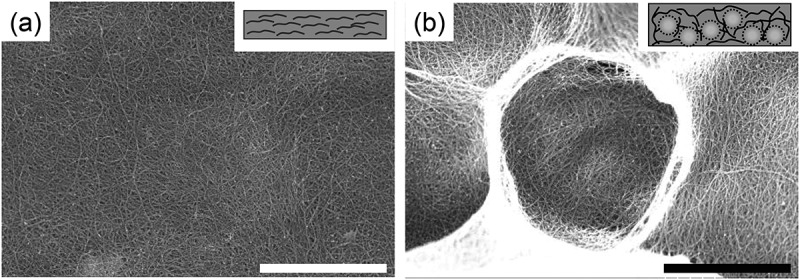
SEM images of (a) SWCNT sheet without PS and (b) SWCNT sheet after the removal of PS particles having a diameter of 3 μm. Scale bar; 2 μm

We added spherical polymer particles as sacrificial orientation aligners of SWCNTs (Scheme 1(b)). We chose polystyrene (PS) particles because of their availability in a uniform size. After fabricating the SWCNT sheet including the PS particles, the particles were removed by heating at 450°C for 1 h. The removal of the PS particles and sodium dodecylbenzenesulfonate (SDBS) used to prepare the sheets was confirmed by thermogravimetric analysis (TGA) of the sheet (Figure S1a). TGA curves of the SWCNT sheet after heating (red line in Figure S1a) showed a one-step weight loss starting at 500°C. The absence of weight loss corresponding to the PS particles at 350°C (black line) and the two-step weight loss of SDBS at 400°C and 600°C indicated the removal of PS and SDBS from the SWCNT sheet. In addition, the absence of the S 2p and Na 1s peaks in the X-ray photoelectron spectroscopy (XPS) narrow scans also supported the removal of the SDBS (Figure S1b and S1c). Such a clear removal of the SDBS only by thermal treatment was one of the advantages of this experiment as a dispersant. Figure 1 shows the scanning electron microscopy (SEM) images of the SWCNT sheet without adding PS particles (Figure 1(a)) and SWCNT sheet with PS particles after the removal of the particles (Figure 1(b)). After the removal of the particles, pore structures corresponding to the size of PS particles (~3 μm) were observed. Such excellent size controllability indicated the formation of a stiff SWCNT network structure around the spherical PS particles, which was preserved after the removal of the particles [9,10]. The pore distribution inside the sheet was monitored by the depth profile of confocal Raman mapping using the characteristic G-band at 1590 cm^−1^ of the SWCNT as an indicator (Figure S2). A pore area with a weak G-band signal (black area) was clearly observed inside the sheet after the removal of the PS particles (Figure S3, lower panels), while the SWCNT sheet without the addition of PS particles showed a uniform distribution of the G-band peak (Figure S3, upper panels). Similar pore formation was also reported using 50–400 nm PS particles as a sacrificial pore source, in which the pore was used to accommodate the charging product of the Li-air rechargeable battery [9,10]. In our system, we found that the spherical orientation of the SWCNT bundle was introduced after the addition of the pore source (Figure 1(b)). Such a spherical orientation renders the orientation of the SWCNT perpendicular to the sheet direction to achieve a three-dimensional orientation. The weight density of the sheets was calculated by dividing the weight of the sheets by the volume of the sheets and was found to be linearly decreased as the amount of PS particles increased (Table S1). Additionally, we recognized that the stiffness of the SWCNT sheets decreased as the pore volume increased, probably because the entanglement of the SWCNTs was weakened with the decrease in the density of the sheet (Figure S4).

**Figure 2.**
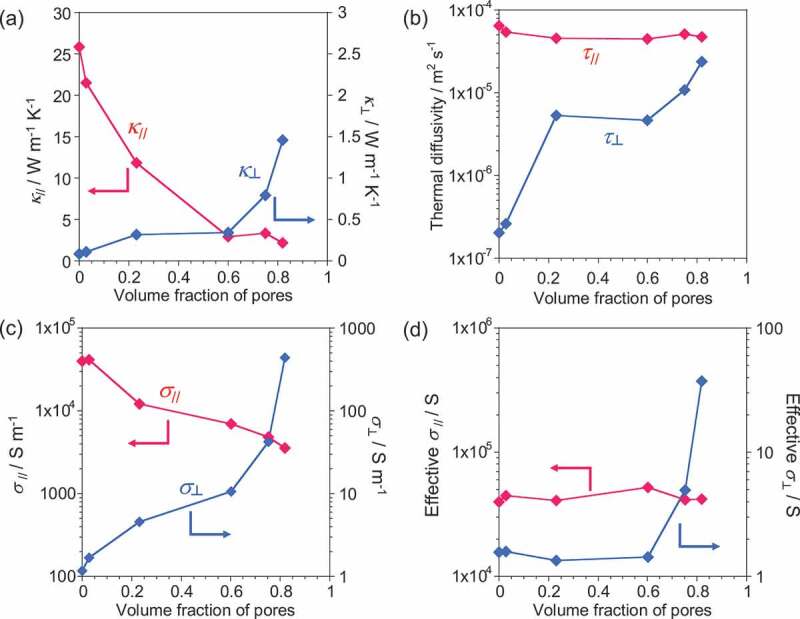
(a) κ, (b) thermal diffusivity, (c) σ, and (d) effective σ of the SWCNT sheets in the in-plane (red line) and through-plane (blue line) directions as a function of the volume fraction of pores

Figure 2(a) shows the dependence of *κ*_//_(red line) and *κ*_⊥_ (blue line) of the SWCNT sheet on pore volume (Table S1). We observed that *κ*_//_decreased but *κ*_⊥_ increased as the pore volume increased. It should be noted that *κ* is the product of the density, thermal diffusivity, and heat capacity (*κ *= ρ × τ × C); thus, theoretically, a decrease in the density will lead to a decrease in *κ* if τ and C are constant. Therefore, the observed increase in *κ*_⊥_ as the pore volume increased is very interesting. As shown in Figure 2(b), the thermal diffusivities in the in-plane direction were almost constant, while those in the through-plane direction dramatically increased as the pore volume increased. Therefore, the increase in *κ*_⊥_ can be attributed to the dramatic increase in the through-plane thermal diffusivity, and it evidenced that the number of spherically oriented SWCNTs increased because of pore introduction. We consider that the percolation network of the thermal conduction path [11] was formed over a pore volume fraction of 0.6. Figure 2(c) shows σ_//_(red line) and σ_⊥_ (blue line) as a function of the volume fraction of the pore. We also observed the decrease of σ_//_as well as an increase of σ_⊥_ as the pore volume increased. Although the SWCNT sheet volume as well as the pore volume increased, the number of SWCNTs was constant. Therefore, we defined “effective σ“ to elucidate only the effect of the anisotropy control of the SWCNT, and it was calculated by multiplying σ with the thickness of the sheet. Interestingly, the effective σ_//_and σ_⊥_ were almost constant below 0.6, while only σ_⊥_ increased over 0.6. This indicates that the through-plane electrical percolation path was connected when 60% volume of the pore was introduced. The result marks a clear contrast to the *κ*_⊥_ showing a linear increase in conductivity with the increase in pore volume, which might have originated from the difference in the conduction mechanisms between *κ* and σ. The results of anisotropy control show that the degree of anisotropy decreased by 1.5 and 8.1 times for *κ*_//_/*κ*_⊥_ and σ_//_/σ_⊥_, respectively, which is close to isotropic conduction.

**Figure 3.**
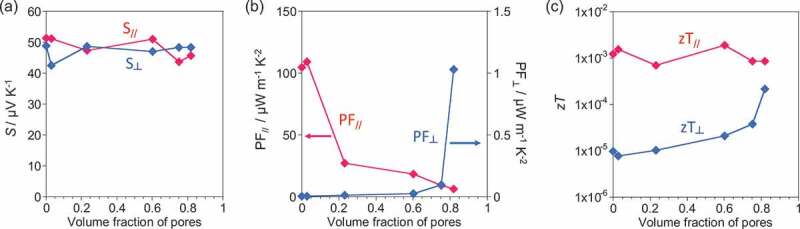
(a) *S*, (b) PF and (c) zT of the SWCNT sheets in the in-plane (red line) and through-plane (blue line) directions as a function of the volume fraction of pores

The Seebeck coefficients of the in-plane (*S*_//_) and through-plane (*S*_⊥_) together with *σ*_//_and *σ*_⊥_ were also measured by applying a temperature difference in the in-plane and through-plane directions, respectively (Figure 3(a)). The Seebeck coefficients, *S*_//_and *S*_⊥_, remained almost unchanged regardless of the pore volume. Because most of the conductance originates from the CNT/CNT junction in the SWCNT network [12], the result suggests that the number of CNT/CNT junctions was almost constant per unit of temperature difference. Based on the above values, we calculated the power factor (PF) and figure-of-merit (zT) of the sheets and found that the PF_//_values decreased as the volume fraction of the PS particles increased (Figure 3(b)), while zT_//_values were almost unchanged (Figure 3(c)). Higher PF_//_and zT_//_compared to PF_⊥_ and zT_⊥_ at any volume fraction clearly suggests that the use of the in-plane direction is preferable for power generation.

### 3.2. Effects of anisotropy control

**Figure 4.**
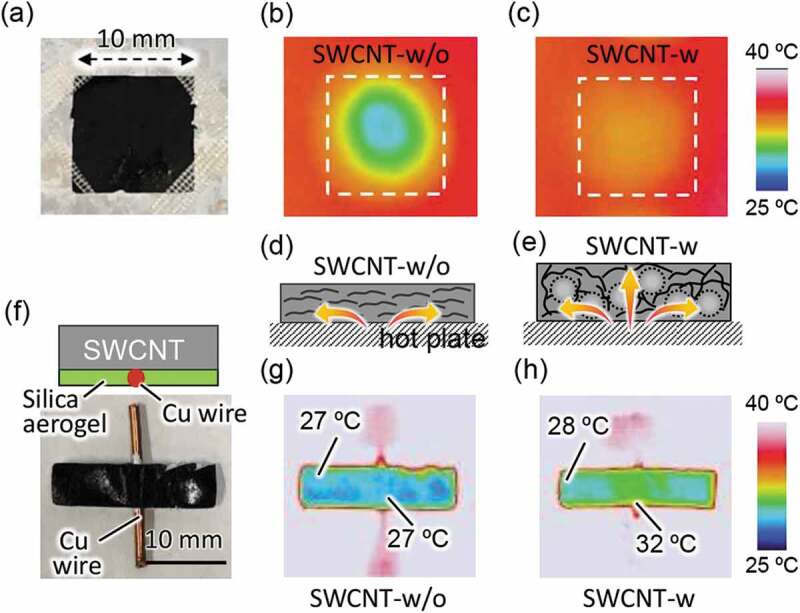
(a) Photo of the SWCNT sheet and thermographs of (b) SWCNT-w/o and (c) SWCNT-w. Schematic illustration of thermal diffusion inside (d) SWCNT-w/o and (e) SWCNT-w. (f) Side-view illustration (upper panel) and top view photo (lower panel) of SWCNT sheet with Cu wire (red) and silica aerogel/poly(vinyl alcohol) composite layer (green). Thermograph of (g) SWCNT-w/o and (h) SWCNT-w

The effects of anisotropy control were studied by monitoring the heating behaviors of the surfaces of the SWCNT sheets with (SWCNT-w) and without anisotropy control (SWCNT-w/o) placed on a hot plate kept at 40°C using an IR camera in the room at 25°C. An SWCNT-w sheet with a volume fraction of 81.9% (thickness: 300 μm) was used because it had the lowest anisotropy in the sample. Interestingly, the thermograph of the SWCNT sheets (Figure 4(a)) after heating for 2 min revealed that the surface temperature of the SWCNT-w (Figure 4(b), thickness of 300 μm) was higher than that of SWCNT-w/o (Figure 4(c), thickness of 30 μm), even though the SWCNT-w was 12 times thicker. The results indicate heat was preferentially delivered to the in-plane direction owing to the large conductivity anisotropy in the SWCNT-w/o sheet (Figure 4(d)), while the SWCNT-w showed homogeneous heat diffusion (Figure 4(e)). That SWCNT-w is favorable for creating a temperature gradient in the in-plane direction.

To investigate the effect of anisotropy control in creating a temperature gradient in the in-plane direction, the center of the SWCNT sheet was locally heated through a Cu wire and the area other than the center was passivated with a thermal insulating layer (TIL) composed of silica aerogel/poly(vinyl alcohol) composite having very low *κ* (*κ* =_ _0.003 W mK^−1^) [7] as shown in Figure 4(f). Figure 4(g,h) display the thermographs of the SWCNT sheets placed on the hot plate (40°C) after heating for 2 min. In SWCNT-w/o (Figure 4(g)), the surface temperature was maintained at 27°C, which was comparable to the room temperature (25°C). In contrast, a clear temperature gradient of 4°C was observed in the SWCNT-w in the in-plane direction (Figure 4(h)). For SWCNT-w/o, the heat was preferentially diffused in the in-plane direction owing to the large conductivity anisotropy and, as a result, the top surface was not sufficiently heated (Figure 4(g)). However, heat was diffused evenly for the SWCNT-w, and the temperature at the central area was heated preferentially, which led to the temperature gradient in the in-plane direction (Figure 4(h)). The results clearly indicate that SWCNT-w is favorable for creating a temperature gradient in the in-plane direction.

**Figure 5.**
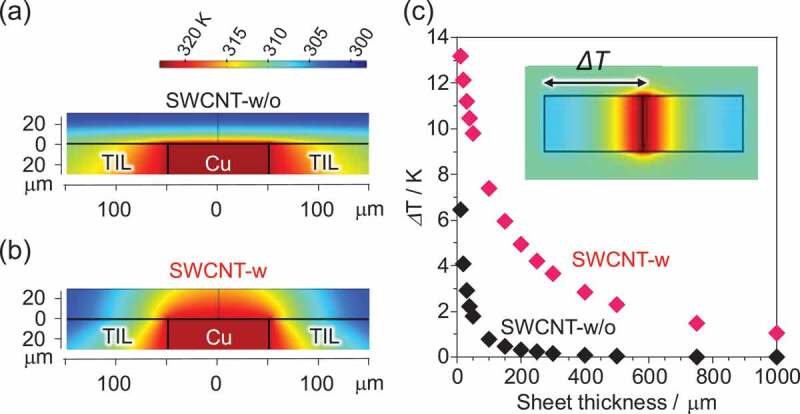
Simulated temperature distribution of (a) SWCNT-w/o and (b) SWCNT-w with a thickness of 30 μm. Hot plate heated at 40°C was placed 1000 μm below the SWCNT sheet. (c) Δ*T* between center and edge (10 mm width) of the SWCNT-w/o (black) and SWCNT-w (red) as a function of sheet thickness

To study the heat diffusion profiles in SWCNT-w and SWCNT-w/o, simulations using COMSOL software were carried out at temperatures of 40°C and 25°C for the hot and cold sides, respectively, and the experimental values of *κ*_//_and *κ*_⊥_ were used in the simulation. Cross-sectional images of the simulated temperature in SWCNT-w/o and SWCNT-w laminated on the Cu (width: 100 μm) and TIL are shown in (Figure 5(a,b)), respectively. The thickness of the sheet was equalized to 30 μm for a fair comparison. Obviously, the lateral direction was preferentially heated in the SWCNT-w/o, while the SWCNT-w was heated homogeneously from the thermally conductive Cu region. The temperature difference between the middle and the end of the SWCNT surface (Δ*T*) is plotted as a function of the sheet thickness (Figure 5(c)). It is clear that Δ*T* decreased dramatically with increasing thickness of the film in the SWCNT-w/o (black), while that of the SWCNT-w decreased gradually (red) and, as a result, a larger Δ*T* was achieved even for a thick film with a thickness of over 100 μm, which is advantageous for a planar-type TE device.

**Figure 6.**
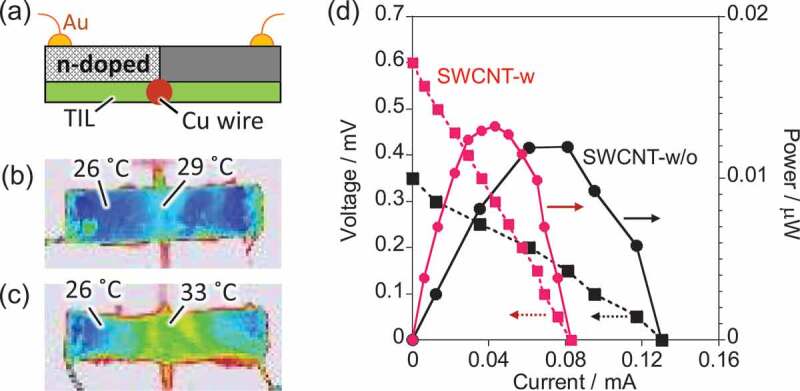
(a) Illustration of the planar-type TE device using SWCNT sheet. Thermograph of the TE device using (b) SWCNT-w/o and (c) SWCNT-w on the hot plate heated at 50°C (d) I–V curves (dotted lines) and power density curves (solid lines) for SWCNT-w/o (black) and SWCNT-w (red)

Finally, to study the superiority of anisotropy control for TE power generation, a single cell structure was fabricated by doping half of the sheet using 2-(2-methoxyphenyl)-1,3-dimethyl-2,3-dihydro-1H-benzo[d]imidazole (*o*-MeO-DMBI) [13] as an n-dopant, and the device was placed on a hot plate (Figure 6(a)). By setting the temperature of the hot plate to 50°C, the temperature gradient between the middle and the end of the SWCNT-w/o was ca. 3°C (Figure 6(b)), while that of the SWCNT-w was 7°C (Figure 6(c)). Figure 6(d) shows the I–V curve (dotted lines) and power density (solid lines) curves of the p-n device for SWCNT-w (red curve) and SWCNT-w/o (black curve), in which the devices showed experimental error of only ±0.8%. We found that, in the in-plane direction, the SWCNT-w and SWCNT-w/o sheets showed open circuit voltage (OCV) and the highest power density of 0.30 mV and 1.32 × 10^−2^ μW cm^−2^ and 0.15 mV and 1.19 × 10^−2^ μW cm^−2^, respectively. This result clearly indicates the effect of anisotropy control for achieving higher power based on higher potential differences created by the larger temperature gradient. Thus, it clearly demonstrates the effect of anisotropy control for a planar-type TE structure. On the other hand, lower short-circuit current for SWCNT-w caused by the higher electrical resistance of the sheet need to be minimized by optimizing the pore volume. Based on the simulation, a much larger temperature gradient can be obtained when the thinner film is fabricated for SWCNT-w (Figure 5(c)). Thus, further optimization of the thickness of SWCNT-w is promising for increasing power density, and such investigations are ongoing.

## 4. Conclusion

SWCNT sheets were prepared in the presence of spherical polymer particles, which were used as orientation aligners. Through anisotropy control, an increase in the SWCNTs oriented in the through-plane direction increased the through-plane thermal and electrical conductivities. Consequently, the large in-plane anisotropy was decreased for both thermal and electrical conductivities. Owing to the anisotropy control, a larger temperature gradient was generated for the SWCNT sheet with anisotropy control, which resulted in a larger OCV as well as a higher maximum power density for the planer-type p-n device compared to those of the SWCNT sheet without anisotropy control. Further improvement of the power density is possible by decrease the thickness of the sheets since a larger temperature gradient can be obtained.

## Supplementary Material

Supplemental Material supporting material

Supplemental Material _ Scheme
