# Supplementary material for: Control of anisotropic conduction of carbon nanotube sheets and their use as planar-type thermoelectric conversion materials
Source: Sci Technol Adv Mater. 2021 Apr 13;22(1):272–9. doi: 10.1080/14686996.2021.1902243 (PMC8049464; doi:10.1080/14686996.2021.1902243)
Supplement: Supplemental Material supporting material [file TSTA_A_1902243_SM5303.docx]

Supporting Information

Control of Anisotropic Conduction of Carbon Nanotube Sheet and Their Use as Planar-type Thermoelectric Conversion Materials

Masamichi Matsumoto, Ryohei Yamaguchi, Keisuke Shima, Masakazu Mukaida, Motohiro Tomita, Takanobu Watanabe, Takao Ishida, and Tsuyohiko Fujigaya*

**Table S1.** List of corresponding values of pore volume (%), density (g cm^−3^), *κ* (W mK^−1^), σ (S m^−1^), S (µV K^−1^), PF (μW mK^−1^), and zT.

**Figure S1.** (a) TGA curves of the SWCNT sheet after heating at 450 ºC (red line), PS particles (blue line), and SDBS (black line) and (b, c) XPS narrow scans of (b) S 2p and (c) Na 1s before (black line) and after (red line) heating at 450 ºC.

**Figure S2.** Raman spectra of the SWCNT sheet after the removal of the PS particles.

**Figure S3.** Confocal Raman mapping of the SWCNT sheet without pores (upper panels) and after the removal of PS particles (lower panels) scanned for the x-y face 5 μm below the surface (left panels) and z direction (right panels). Scale bars; 5 μm.

**Figure S4.** Plot of the compression rupture strength of SWCNT sheets having different pore volumes.
