# Supplementary figures and images for: Control of anisotropic conduction of carbon nanotube sheets and their use as planar-type thermoelectric conversion materials
Source: Sci Technol Adv Mater. 2021 Apr 13;22(1):272–9. doi: 10.1080/14686996.2021.1902243 (PMC8049464; doi:10.1080/14686996.2021.1902243)

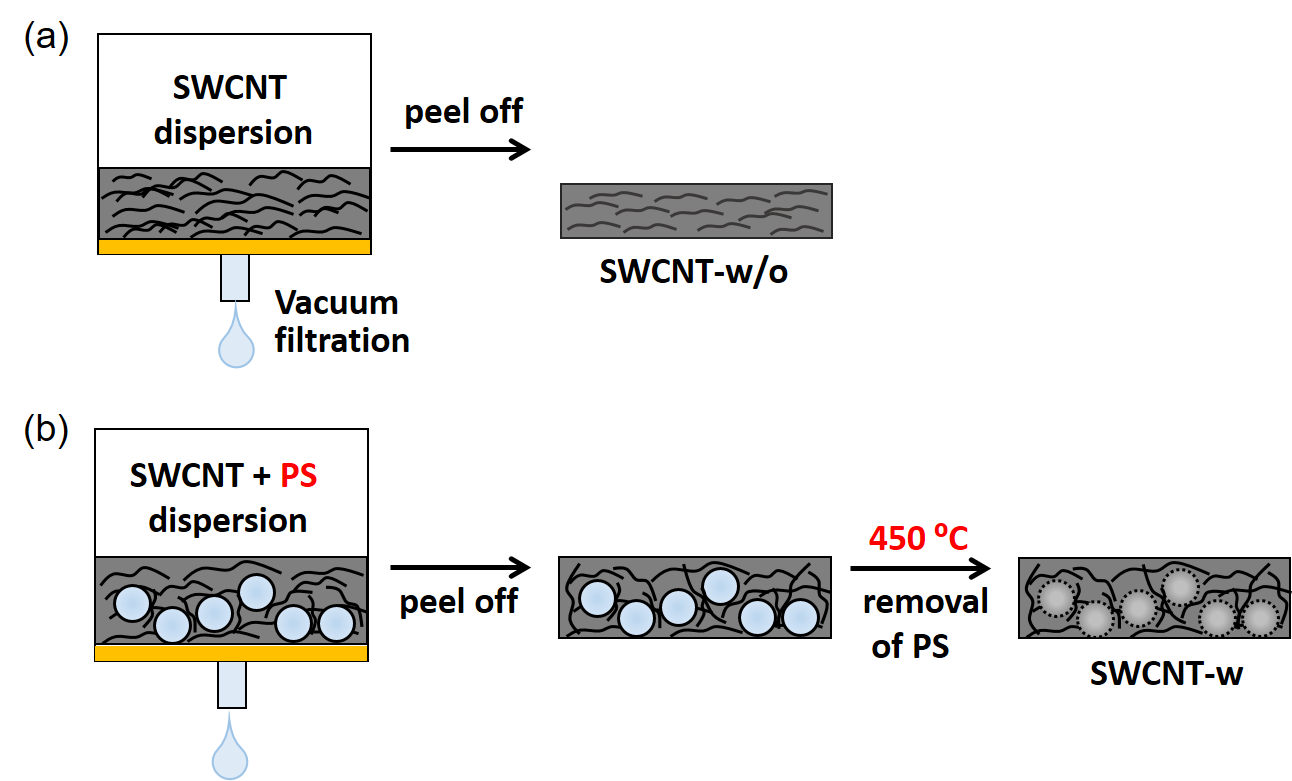

Supplement: Supplemental Material _ Scheme [file TSTA_A_1902243_SM5296.tif]
